# Societal costs of older adults with low back pain seeking chiropractic care: findings from the BACE-C cohort study

**DOI:** 10.1186/s12998-024-00553-0

**Published:** 2024-11-06

**Authors:** Esther T. Maas, Brenda L. van der Vossen, Johanna M. van Dongen, Alan D. Jenks, Sidney M. Rubinstein

**Affiliations:** 1 Department of Health Sciences, Faculty of Science, The Amsterdam Movement Sciences Research Institute, Vrije Universiteit Amsterdam, Van Der Boechorststraat 3, 1081 BT Amsterdam, The Netherlands; 2 Capilano University, 2055 Purcell Way, North Vancouver, BC V7J 3H5 Canada

**Keywords:** Low back pain, Older adults, Societal costs, Prediction

## Abstract

**Background:**

To describe the societal costs during one year of follow-up among older adults seeking chiropractic care due to a new episode of low back pain (LBP), and to determine what factors predict high societal costs in this population.

**Methods:**

Prospective cohort study, within chiropractic private practices (n = 38) in the Netherlands. 223 people ≥ 55 years of age with a new episode of LBP seeking chiropractic care participated. The primary outcome was total societal costs. High societal costs were defined as patients with costs in the top 20th percentile. The final prediction models were obtained using forward selection. Results were presented for the total population and stratified for retirement status. The model’s prognostic accuracy (Hosmer–Lemeshow X^2^, Nagelkerke’s R^2^) and discriminative ability [area under the receiver operating curve (AUC)] were assessed, and the models were internally validated using bootstrapping.

**Results:**

The mean total annual societal cost per patient was €5297 [95% confidence interval (CI): 4191–6403]. The biggest cost driver was presenteeism (65% of total costs), and costs were higher among non-retired participants (€7759; 95% CI 6047–9470) than retired participants (€1892; 95% CI 1088–2695). In the total population, younger age [odds ratio (OR): 0.87 for each additional year; 95% CI 0.80–0.95], being male instead of female (OR 2.96; 95% CI 1.19–7.44), less alcohol intake (OR 0.49; 95% CI 0.20–1.19), working instead of retirement (OR 9.37; 95% CI 1.83–48.04), and more disability at baseline (OR 1.08; 95% CI 1.00–1.16) were found to be predictive of high societal costs. Working was found to be the strongest predictor for high societal costs. After internal validation, the model’s fit was good, it’s explained variance was moderate (28%) and their AUCs could be interpreted as moderate (0.85). For non-pensioners, the same predictive factors were identified as for the entire population. The costs for the retired participants showed too little variation to be able to predict high costs.

**Conclusions:**

This study estimated the mean total annual societal cost of older adults seeking chiropractic care due to a new episode of LBP at €5297 (95% CI 4191–6403).These costs were mainly due to high levels of presenteeism, and extensively differed based upon work status.

**Supplementary Information:**

The online version contains supplementary material available at 10.1186/s12998-024-00553-0.

## Introduction

Low back pain (LBP) is a major cause of sickness absence, work disability, reduced productivity, and early retirement [1, 2]. LBP and its related sickness absence are associated with significant costs for individuals and society, which are expected to increase during the upcoming decades due to the aging population [3]. Despite this, there is limited information about the clinical trajectory and societal ramifications of LBP in older adults compared to their younger counterparts.

LBP ranks among the most prevalent complaints encountered in primary care. For primary care providers (e.g. physiotherapists, chiropractors) it is important to identify risk factors that are associated with higher costs in older adults with LBP. If such factors are identified and known, targeted interventions can be offered before incurring substantial avoidable costs and a decline in health status. The Commonwealth Fund (2012) underlines the importance of addressing high-cost users with chronic conditions [4]. A recent study in the Netherlands identified factors associated with high societal costs among people with chronic LBP, including poor physical health, high functional disability, low health-related quality of life, high impact of pain experience, and non-Dutch nationality [5]. However, older people with LBP might have different cost-patterns in comparison to younger people with LBP (e.g. due to their retirement and/or co-morbidities). Therefore, it is important to identify the possible risk factors for high costs in older adults with LBP as well.

To improve use of scarce resources and thus to reduce the burden on our healthcare systems, research has highlighted the importance of monitoring and understanding healthcare utilisation and costs related to LBP for older adults [6]. Improving our understanding of this population and the course of their LBP and costs related to their LBP may provide valuable input for studies regarding the effectiveness and cost-effectiveness of chiropractic care.

This study investigates factors associated with high costs of LBP in older adults using the BAck Complaints in the Elders-Chiropractic (BACE-C) study, an international cohort study dedicated to examining back complaints in older patients in primary care [4]. The BACE-C study is uniquely positioned in the chiropractic care setting due to limited information about the clinical course of LBP in older adults, especially those seeking chiropractic care. The primary objectives of the BACE-C study were to examine the one-year clinical course of pain intensity and improvement rates of LBP in individuals aged 55 years and older who visit a chiropractor for a new episode of LBP. Cost data were also collected within the same population, offering an opportunity to study the costs related to LBP in this demographic—an essential step considering the historical underrepresentation of older people in back pain research [4].

The primary objective of this study is to study societal costs over one year incurred by older adults in the Netherlands who consult a chiropractor for a new episode of LBP. The second objective is to identify predictive factors associated with high societal costs in older adults with LBP.

## Methods

This study is reported according to the consensus-based checklist for the critical appraisal of cost-of-illness studies [7] (Appendix 1). A study protocol of the BACE-C has been published [8]. Ethics approval has been obtained by the Medical Ethics Committee of the Vrije University Medical Center, the Netherlands ethics number 2017–618.

### Study design and setting

The Back Complaints in Elderly-Chiropractic (BACE-C) study was designed as an international, multi-centred prospective cohort study. The BACE-C is part of the international BACE consortium [9]. For this study, only Dutch participants were included, and recruited from 38 private practices of chiropractors in the Netherlands. All questionnaires were completed electronically. Follow-up measurements were collected at 2 and 6 weeks, 3, 6 and 9 months, and one year after the first treatment. The data collected at 2 and 6 weeks were not used in this study as data on costs were not included at those follow-up moments.

### Participants

*Inclusion criteria*: Adults aged 55 and older who consulted a chiropractor for a new episode of LBP, meaning LBP for the first time, or those adults who have not been to a chiropractor in the previous six months were eligible for inclusion. This is independent of whether they have seen another type of healthcare provider for the current episode. All low back complaints, with pain in the region from the thoracolumbar 12th rib junction to the first sacral vertebrae, including pelvic pain and pain referral to the leg(s) were eligible. Chiropractors who are licensed and currently work in clinical practice were asked to participate.

*Exclusion criteria:* Patients with cognitive disorders, a suspected tumour, fracture, infection or any other potential red flag or condition considered to be a contraindication for chiropractic care were excluded.

### Outcome measures

The primary outcome for the first objective was total annual societal costs, and total societal costs separated by cost category and by three-month time frames. Societal costs were measured using 3-monthly retrospective cost questionnaires throughout the 1-year study period (i.e. administered at 3-, 6-, 9- and 12-month follow-up), which is included in Appendix 2. The self-administered cost questionnaires included measures of health care utilisation, informal care, unpaid productivity, presenteeism and absenteeism. Health care utilisation included primary care (e.g. general practitioner care, manual therapy, physical therapy, exercise therapy) and secondary care (e.g. diagnostic and therapeutic interventions, hospitalisation) [10]. Data from the updated Dutch Manual of Costing were used to value costs of common health care services [11]. For less common healthcare services, hospital accounting records and/or prices of professional organisations were used. Informal care and unpaid productivity were valued using the recommended Dutch shadow price of €15.29 per hour [12]. Absenteeism and presenteeism from paid employment was measured using the Productivity and Disease Questionnaire [13], and was valued in accordance with the friction cost approach using hourly productivity costs of males and females [14]. The friction cost approach assumes that production losses are confined to the period needed to replace a sick worker (i.e. friction period), which is currently assumed to be 12 weeks in the Netherlands [14]. All costs were expressed in Euros 2021.

The primary outcome for objective 2 was having high societal costs (yes/no). Having high societal costs was defined as patients with costs in the top 20th percentile, which is consistent with previous studies [15–17]. For this study, the 20th percentile for societal costs was, therefore, assumed to be appropriate and feasible due to the relatively small sample size.

### Potential predictive factors

Potential predictive factors for high societal costs were based on previous literature [15, 18], and were measured at baseline:

- LBP intensity, measured on an 11-point numeric rating scale (NRS) [19], ranging from 0 ‘no pain ‘to 10 ‘the worst pain ever’.
- Back-specific functional status: Roland Morris Disability Questionnaire (RMDQ) [20].
- Global perceived effect (GPE), measured on a 7-point scale, ranging from ‘completely recovered’ to ‘worse than ever’ [21].
- Sociodemographic characteristics (i.e. age, gender, marital status, education level, height and weight (for BMI), ethnicity including parental ethnicity).
- Physical activity, measured with the International Physical Activity questionnaire.
- Other lifestyle variables: smoking measured by pack/years, alcohol use measured by the short version of the AUDIT [22], and sleeping habits measured by the short version of the Pittsburgh Sleep Quality Index)[23].
- Comorbidities, measured with the Self-administered Comorbidity Questionnaire [24].
- Indicator screening tool (STarT Back) for poor outcome [25].
- Health-Related Quality-of-life, measured with the EQ-5D-5L [26].

### Missing data

Missing data were visually explored, and missingness at random was assumed. Missing data were handled using multivariate imputation by chained equations to avoid possible bias due to selective drop-out of participants, which might influence the results when conducting a complete-case analysis [27]. The imputation model included sex, smoking, marital status, age, BMI, back pain complaint history, education, treatment expectations, and relevant baseline effect measure values. For each measurement moment, each cost category was imputed (primary care costs, secondary care costs, unpaid productivity, informal care, absenteeism, and presenteeism). Ten complete data sets were created so that the loss of efficiency would be smaller than 5%. Complete datasets were analysed as outlined below, after which pooled estimates were calculated using Rubin’s rules [27].

### Statistical analyses

Descriptive statistics (frequency counts and proportions) were used to describe the study cohort, and their related costs for the full year. Costs were described in euros with means (95% confidence intervals (CI)). Similarly, the total societal costs were presented separately for 3-month time frames.

The prediction model was constructed using multivariable logistic regression analysis. Univariate logistic regression was performed to pre-select variables based on statistical significance (*p* < 0.20). This was done because manual forward selection was used to obtain the final predictive factors with a *p* < 0.10. Variables with the lowest p-value were included in the model one by one and the analyses were re-run until only variables with a *p* < 0.10 constituted the model. A *p* < 0.10 was used to ensure that predictions are accurate, whilst preventing type-1 errors caused by overfitting [28]. We opted for manual forward selection because of the small sample size.

The overall performance and predictive ability of the model were tested using Nagelkerke R^2^ [28, 29]. The other performance measures included the area under the receiver operating characteristics curve (AUC) to measure the final model’s discriminative value [28, 29].

To adjust for the fact that the model was developed and tested in the same population, which typically causes regression coefficients and performance measures to be overestimated (i.e. overfitting), bootstrapping was used to internally validate the model [29].

Descriptive statistics and prediction models were performed in Stata 17 (Stata Corp LP, College Station, TX). The internal validation was performed using R (version 2023.09.1 + 494).

### Subgroup analyses

Absenteeism and presenteeism are large cost drivers for workers with LBP [30]. For this reason, separate analyses were performed based on retirement status: for pensioners (who do not have costs related to absenteeism and presenteeism), and participants who are active in the workforce (non-pensioners).

### Post-hoc analyses

Based on the findings that NRS and costs seem to show a similar pattern, a Pearson’s correlation test between pain severity (Numeric Rating Scale (NRS) 0–10) and total societal costs as well as health-related functioning and total societal costs at three months was done to statistically test these descriptive statistics.

## Results

### Participant characteristics

**Fig. 1:**
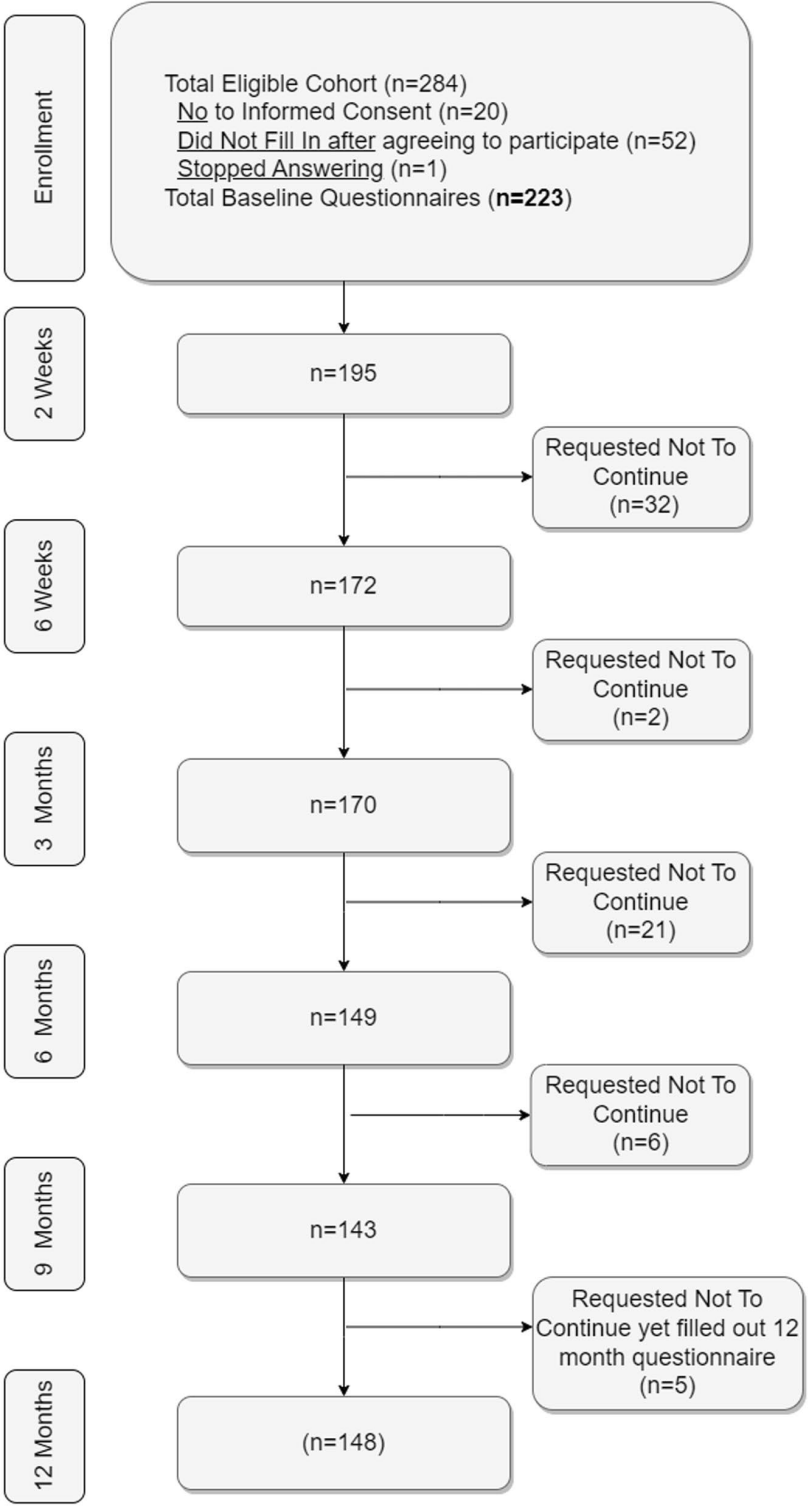
Flowchart of BACE-C cohort

Figure 1 shows the flow chart of included participants in the BACE-C study, in which 284 people were eligible for participation. After exclusion of participants that did not consent or did not fill in any questionnaires, despite agreeing to do so, a total of 223 people were included in the study. The percentage of missing data ranged from 0 to 28% for included baseline variables, for which one variable (feelings of depression) was an extreme outlier in this case; i.e. 148 participants had complete data.

Table 1 shows the characteristics of all participants. The mean age of the participants was 66.4 years (standard deviation (SD) 7.6 years), of whom 42.3% described themselves as a pensioner. Of the 129 non-pensioners, 100 (77%) were younger than the retirement age of 67 years in the Netherlands. Of the 90 pensioners, 17 (19%) was younger than 67 years of age. The mean BMI was 26.6 (SD 4.7) and 82.1% was married or living with a partner. There was a slightly smaller proportion of females (46.6% vs 53.4% males). Of the participants, 88.6% did not smoke, and 54.6% drank alcohol on average more than twice per week. In total, 40.8% had a low-level education, 21.5% had moderate level education, and 37.7% had a high level of education. Almost all (94.6%) participants had the Dutch nationality. The top five comorbidities is neck/shoulder symptoms (55.2%), feet problems (28.3%), hip/knee arthritis (27.8%), high blood pressure (26.5%) and headache/migraine (17.9%). The median symptom duration was 122 days (interquartile range (IQR): 21–1095 days).

**Table 1:** Characteristics of all participants, according to societal costs (high vs. low), and according to retirement status (non-pensioner vs. pensioner)

	All participants (n = 223)	Missing n(%)	High costs (n = 21)*	Low costs (n = 202)	Non-pensioner (n = 129)	Pensioner (n = 90)
Age (years) [mean(SD)]	66.4 (7.6)	0 (0%)	60.4 (4.0)	66.9 (7.6)	63.0 (6.9)	71.3 (5.9)
Gender—female [n(%)]	104 (46.6%)	0 (0%)	7 (33.3%)	97 (48.0%)	71 (55.0%)	30 (33.3%)
BMI [mean(SD)]	26.6 (4.7)	0 (0%)	27.8 (4.0)	26.5 (2.7)	26.7 (4.6)	26.4 (4.8)
Smoking—yes [n(%)]	23 (11.4%)	21 (9%)	1 (4.8%)	22 (12.2%)	18 (15.0%)	5 (6.1%)
*Average alcohol consumption [n(%)]*						
Never	30 (14.8%)	20 (9%)	1 (4.5%)	29 (15.9%)	21 (17.4%)	9 (11.0%)
Once per month or less	28 (13.8%)	20 (9%)	4 (19.1%)	24 (13.2%)	18 (14.9%)	10 12.2%)
24 times per month	34 (16.8%)	20 (9%)	3 (14.3%)	31 (17.0%)	22 (18.2%)	12 14.6%)
2–3 times per week	46 (22.6%)	20 (9%)	7 (33.3%)	39 (21.4%)	30 (24.8%)	16 (19.5%)
4 > 4 times per week	65 (32.0%)	20 (9%)	6 (28.6%)	59 (32.4%)	40 (24.8%)	35 (42.7%)
*Educational level [n(%)]*						
Low	91 (40.8%)	0 (0%)	7 (33.3%)	84 (41.6%)	60 (46.5%)	29 32.2%)
Moderate	48 (21.5%)	0 (0%)	6 (28.6%)	42 (20.1%)	32 (24.8%)	15 (16.7%)
High	84 (37.7%)	0 (0%)	8 (38.1%)	76 (37.6%)	37 (28.7%)	46 (51.1%)
*Marital status [n(%)]*						
Single	32 (14.4%)	0 (0%)	3 (14.3%)	29 (14.4%)	16 (12.4%)	16 17.8%)
Married/living together	183 (82.1%)	0 (0%)	18 (85.7%)	165 (81.7%)	108 (83.7%)	71 78.9%)
LAT	8 (3.6%)	0 (0%)	0 (0.0%)	8 (3.9%)	5 (3.9%)	3 (3.3%)
*Nationality [n(%)]*						
Dutch	211 (94.6%)	0 (0%)	21 (100%)	190 (94.1%)	120 (93%)	87 (96.7%)
Non-Dutch	12 (5.4%)	0 (0%)	0 (0%)	12 (5.9%)	9 (7%)	3 (3.3%)
*Country of origin father [%/%]*						
Dutch/non-Dutch	89.2%/10.8%	0 (0%)	95.2%/4.8%	88.6%/11.4%	89.1%/10.9%	88.9%/11.1%
*Country of origin mother [%/%]*						
Dutch/non-Dutch	92.4%/7.6%	0 (0%)	95.2%/4.8%	92.1%/7.8%	91.5%/8.5%	93.3%/6.7%
*Employment*						
(Self-)employed	98 (43.9%)	0 (0%)	18 (18.4%)	80 (81.6%)	NA	NA
Pensioner	90 (40.4%)	0 (0%)	0 (0.0%)	90 (100%)	NA	NA
Other (e.g. voluntary work)	31 (15.7%)	4 (1.8%)	3 (8.6%)	28 (91.4%)	NA	NA
Feelings of depression [n(%)]	10 (5.5%)	63 (28.0%)	0 (0.0%)	10 (6.3%)	6 (5.6%)	4 (5.4%)
Symptom duration in days (median (IQR)	121 (21–1095)	26 (12)	122 (28–1095)	61 (14–730)	122 (28–1095)	117 (21–730)
Comorbidities (N (%))**						
Neck/shoulders symptoms	101 (55.2%)	0 (0%)	11 (52.3%)	90 (44.6%)	59 (45.7%)	42 46.5%)
Feet problems	51 (28.3%)	0 (0%)	6 (28.6%)	45 (22.3%)	29 (22.5%)	22 24.4%)
Hip/knee arthritis	49 (27.8%)	0 (0%)	5 (23.8%)	44 (21.8%)	24 (18.6%)	25 27.8%)
High blood pressure	49 (26.5%)	0 (0%)	6 (28.6%)	43 (21.3%)	29 (22.4%)	20 (22.2%)
Headache/migraine	32 (17.9%)	0 (0%)	3 (6%)	29 (14.4%)	24 (18.6%)	8 (8.9%)
QALY [mean (SD)	0.4 (0.4)	23 (10)	0.3 (0.4)	0.4 (0.4)	0.3 (0.4)	0.4 (0.4)
Pain intensity [mean(SD)]	6.0 (2.2)	22 (10)	6.6 (2.1)	5.9 (2.2)	6.0 (4.6)	6.0 (2.1)
RMDQ [mean(SD)]	9.6 (5.8)	22 (10)	10.9 (6.5)	9.5 (5.7)	9.7 (5.9)	9.6 (5.6)

*BMI *Body Mass Index, *QALY *Quality Adjusted Life Years, *RMDQ*  Roland Morrison Disability Index, *SD *Standard Deviation

*Participants categorized in high costs group were less than 20% due to missing data. Note: Percentages have been rounded off, hence values a bit less than 100% and a bit more than 100%

The group of participants in the 80% lowest costs (n = 202) had overall similar characteristics to the group of all participants (Table 1). In the group of participants with the 20% highest healthcare costs (n = 21), 66.7% were male, the average age was 60.4 years (SD:4.0), the average BMI was (27.8 (SD:4.0), and most people were non-smokers (87.8%). Back-specific functional status was higher (10.9, SD:6.5), as was the pain intensity (6.6, SD:2.1) in this group compared to the lowest cost group.

Of the 223 included participants, 90 participants were retired, 129 were not pensioned of which 98 participants had paid work. Of the working participants, 21 have experienced one or more days of sickness absence within the year after inclusion [median 17 days (IQR: 3–20)]. Eight of these participants exceeded the friction cost period. At each measurement moment, between 9 and 13 participants indicated to perform 100% at work. All other participants have indicated a level of presenteeism. Pensioners were on average 71.3 years(SD:5.9), 66.7% male, had a high percentage of non-smokers (93.9%), and had a high percentage of participants with an average alcohol consumption of four times per week or more (42.7%). Most pensioners had followed high level education (51.1%) and were married (78.9%).

### Total annual societal costs

Table 2 gives an overview of the participants’ aggregate and disaggregate costs. The mean total annual societal costs were €5297 (95% CI 4191–6403). Presenteeism was the biggest cost driver, accounting for 66% of total societal costs. The cost distribution is shown in Appendix 3.

**Table 2:** Overview of the costs in Euros for people with low back pain in the BACE-C study (mean (95% Confidence Interval*))

		Medication costs	Primary healthcare costs	Secondary Healthcare costs	Absenteeism costs	Presenteeism costs	Unpaid productivity costs	Informal care costs	Total societal costs
All participants (N = 223)	0–3 months	95 (39–151)*	131 (97–164)	72 (24–119)	96 (7–187)	1263 (874–1652)	32 (16–48)	66 (17–115)	1755 (1325–2188)
	3–6 months	66 (17–116)	90 (63–118)	84 (50–117)	234 (52–418)	683 (422–945)	24 (9–40)	39 (10–69)	1220 (850–1594)
	6–9 months	180 (38–321)	75 (51–98)	86 (44–130)	75 (0–178)	824 (523–1124)	23 (11–36)	7 (0–20)	1270 (913–1625)
	9–12 months	76 (5–146)	50 (30–71)	97 (36–159)	54 (7–101)	705 (462–948)	155 (0–364)	24 (0–53)	1162 (823–1502)
	Total 12 months**	**417 (183–651)**	**346 (283–411)**	**339 (230–449)**	**459 (128–766)**	**3475 (2523–4427)**	**234 (25–446)**	**136 (31–212)**	**5297 (4191–6403)**
All participants—high costs (N = 21)	0–3 months	7 (0–14)	162 (69–255)	82 (0–214)	360 (0–890)	6568 (4131–9008)	2 (0–7)	149 (0–424)	7330 (4628–10,033)
	3–6 months	63 (0–173)	102 (42–161)	38 (0–91)	1381 (0–2813)	3975 (3416–4533)	18 (0–41)	96 (0–214)	5672 (2941–8403)
	6–9 months	54 (0–162)	80 (29–131)	137 (4–270)	565 (0–1678)	4833 (2671–6994)	9 (0–24)	70 (0–217)	5747 (3336–8159)
	9–12 months	56 (0–163)	72 (25–119)	58 (0–143)	263 (0–574)	3991 (2586–5396)	16 (0–43)	52 (0–145)	4508 (2915–6102)
	**Total 12 months**	**181 (0–505)**	**415 (284–545)**	**314 (65–564)**	**1904 (244–3564)**	**19,368 (14,889–23,846)**	**45 (7–84)**	**367 (6–728)**	**22,595 (17,255–27,935)**
All participants—low costs (N = 202)	0–3 months	106 (87–124)	128 (121–136)	68 (56–79)	77 (49–105)	782 (697–867)	36 (31–40)	59 (45–72)	1169 (1075–1263)
	3–6 months	40 (30–50)	87 (81–93)	88 (81–95)	20 (4–35)	181 (153–210)	25 (21–29)	25 (18–31)	462 (423–500)
	6–9 months	111 (89–134)	74 (69–80)	84 (73–94)	4 (1–9)	159 (131–186)	27 (24–30)	0 (0–0)	494 (458–530)
	9–12 months	55 (38–72)	47 (43–51)	108 (94–121)	5 (0–9)	155 (130–181)	68 (40–95)	9 (5–14)	427 (391–463)
	**Total 12 months**	**427 (347–508**	**337 (322–353)**	**350 (321–379)**	**203 (148–258)**	**1820 (1644–1995)**	**262 (188–336)**	**114 (91–137)**	**2035 (1937–2133)**
Non-pensioners (N = 129)	0–3 months	92 (30–155)	132 (90–174)	51 (1–99)	168 (12–323)	2162 (1532 – 2793)	29 (8–50)	48 (0–101)	2681 (1991–3372)
	3–6 months	62 (1–123)	76 (44–108)	85 (38–132)	406 (91–721)	1175 (740–1610)	23 (5–40)	26 (0–57)	1852 (1238–2466)
	6–9 months	184 (0–396)	74 (48–101)	67 (19–115)	130 (0–308)	1416 (919–1914)	18 (1–35)	11 (0–34)	1902 (1321–2482)
	9–12 months	70 (0–164)	44 (20–69)	65 (0–137)	94 (13–174)	1185 (793–1576)	52 (0–124)	12 (0–36)	1521 1082–1961)
	**Total 12 months**	**409 (137–680)**	**326 (247–406)**	**268 (148–387)**	**599 (225–974)**	**5939 (4427–7449)**	**121 (44–198)**	**97 (23–171)**	**7759 (6047–9470)**
Pensioners (N = 90)	0–3 months	103 (0–211)	131 (87–174)	67 (25–110)	0 (0–0)	0 (0–0)	38 (14–61)	95 (4–186)	464 (269–659)
	3–6 months	75 (0–167)	106 (67–145)	78 (41–115)	0 (0–0)	0 (0–0)	27 (3–51)	42 (0–84)	338 (207–469)
	6–9 months	174 (9–339)	72 (42–102)	116 (49–183)	0 (0–0)	0 (0–0)	32 (13–51)	0 (0–0)	406 (217–594)
	9–12 months	87 (0–196)	60 (32–89)	136 (54–217)	0 (0–0)	0 (0–0)	310 (0–825)	42 (0–107)	684 (134–1234)
	**Total 12 months**	**439 (3–876)**	**370 (281–458)**	**398 (237–558)**	**0 (0–0)**	**0 (0–0)**	**407 (0–921)**	**179 (29–329)**	**1892 88–2695)**

*Based on the study of Nixon et al. (2010), using Monte Carlo simulations, the authors showed that, even when data were highly skewed, both a normal-based method (e.g. OLD) and non-parametric bootstrapping accurately estimated the true standard errors (SEs), and hence 95%Cis, when sample sizes were moderate to large (*n* > 50), also gave good estimates for small data sets with low skewness

**Total values are depicted in bold font. 95% CI: Confidence Interval. Costs are presented in euros 2021

Participants were categorized as high-cost user (highest 20%) if their total annual societal costs were ≥ €8694. The average total societal cost in the high-cost group was €22,595 (95% CI 17,255–27,935) and for the low-cost group €12,035 (95% CI 1937–2133).

Table 2 shows the average total societal costs for each follow-up period and aggregated for one year of follow-up. It shows relatively high presenteeism costs in the first three months, which slightly declines and stabilizes after three months. It is noteworthy that the high presenteeism costs for the first three months then declining and stabilising for the rest of the year follows a similar pattern to the NRS for pain intensity over the year. The NRS declines from 5.55 (scale 0–10) to 2.27 after three months and remains stable around 2 on a scale 0–10 (Appendix 4). Most other costs categories remain stable over the period of one year (See Appendix 4). This pattern is found in all groups (high- versus low-cost; and pensioners versus non-pensioners).

Table 2 also gives an overview of the average societal costs the pensioners and non-pensioners with low back pain in the BACE-C study made in one year. Total mean annual societal costs were 7759 (95% CI 6047–9470) for the non-pensioners. All pensioners were categorised in the low-cost group, largely due to the lack of absenteeism and presenteeism costs. The total mean societal costs for the group of low costs pensioners were 2035 (95% CI 1937–2133).

### Prediction of total societal costs

Using the top 20th percentile of societal costs as an outcome, the predictive factors for high societal costs in the total population were younger age (OR 0.87 for each additional year; 95% CI 0.80–0.95), being male instead of female (OR 2.96; 95% CI 1.19–7.44), lower alcohol intake (OR 0.49 for each increase category of alcoholic intake; 95% CI 0.20–1.19), working instead of retirement (OR 9.37; 95% CI 1.83–48.04) and more back-related functional disability at baseline (OR 1.08 for each additional point on the RMDQ; 95% CI 1.00–1.16) (Table 3).

**Table 3:** Multivariate model using the top 20th percentile of societal costs as an outcome (total population)

	Coefficient (regression)	SE (of regression coefficient)	*p* value	Odds ratio	95% CI
Age*	− 0.14	0.04	0.002	0.87	0.80–0.95
Gender*	1.09	0.47	0.020	2.96	1.19–7.44
Alcohol intake*	− 0.72	0.45	0.114	0.49	0.20–1.19
Retirement status*	2.24	0.83	0.007	9.37	1.83–48.04
Back-related functioning*	0.07	0.4	0.040	1.08	1.00–1.16

*Age: Continuous. Gender: 0-female, 1-male. Alcohol intake: alcoholic drinks per day (1–10). Retirement: 0 = Pensioner; 1 = non-pensioner. Back-related functioning: functioning at baseline (Roland Morrison Disability Questionnaire 0–24)

The Hosmer–Lemeshow statistic was not significant (X^2^ = 6.27 p = 0.62), indicating that the model’s overall fit was good. The model explained 28.2% (Nagelkerke’s R^2^) of the variation in the outcome (i.e. high societal costs) and the model’s AUC was 0.86 (Table 5).

**Table 4:** Multivariate model using the top 20th percentile of societal costs as an outcome (non-pensioners)

	Coefficient (regression)	SE (of regression coefficient)	*p* value	Odds ratio	95% CI
Gender*	1.28	0.56	0.023	3.60	1.19–10.87
Alcohol intake*	− 1.07	0.59	0.07	0.34	0.11–1.10
Age*	− 0.11	0.05	0.028	0.89	0.80–0.99
Back-related functioning*	0.10	0.05	0.025	1.10	1.01–1.21

^*^Age: Continuous. Gender: 0-female, 1-male. Alcohol intake: alcoholic drinks per day (1–2, 2–4, 5–6, 7–9). Back-related functioning: functioning at baseline (Roland Morrison Disability Questionnaire 0–24)

After internal validation, the model’s explained variance reduced to 35.7 and the AUC to 0.84.

### Sensitivity analysis: prediction of societal costs for the non-pensioners

Using the top 20th percentile of societal costs as an outcome in the non-pensioners, the predictive factors for high societal costs were younger age (OR 0.89 for each additional year; 95% CI 0.80–0.99), being male (OR 3.60; 95% CI 1.19–10.87), lower alcohol intake (OR 0.34 for each increase category of alcoholic intake; 95% CI 0.11–1.10) and more back-related functional disability at baseline (OR 1.10 for each additional point on the RMDQ; 95% CI 1.01–1.21) (Table 4).

The Hosmer–Lemeshow statistic for the total sample was not significant (X^2^ = 6.28 p = 0.83), indicating that the model’s overall fit was good. The model explained 14.9% (Nagelkerke’s R^2^) of the variation in the outcome (i.e. high societal costs) and the model’s AUC was 0.77 (Table 5).

**Table 5:** Summary of performance of predictive models

	Hosmer Lemeshow test (*p* value)	Area under ROC curve	Nagelkerke R^2^
Total sample (N = 223)	6.271 (0.62)	0.8578	0.2818
Non-pensioners (N = 129)	6.282 (0.83)	0.7667	0.149

After internal validation, the model’s explained variance reduced to 26.5 and the AUC to 0.77.

### Prediction of societal costs for the pensioners

Due to the low total societal costs and lack of variability in healthcare and informal care costs, we did not find any predictive factors for high costs for the pensioners. An overview of the univariate analyses, showing none of the potential predictive factors had a *p* value < 0.02 is included in Appendix 5.

### Post-hoc analysis: Correlation between pain severity and costs at three months

A Pearson’s correlation test between pain severity, and total costs at three months, showed a correlation of 0.10 between pain severity and total societal costs (no correlation), and a correlation of 0.12 between pain severity and healthcare costs.

## Discussion

### Main findings

The first aim of this study was to estimate the annual societal costs made by older adults with LBP seeking chiropractic care due to a new episode of LBP. The mean total annual societal costs per patient was €5297 (95% CI 4191–6403). Most of these costs comprised of presenteeism and absenteeism costs. Participants categorised in the 20% highest costs group had an average of €22,595 (95% CI 17,255–27,935), whereas participants categorised in the lowest 80% cost group had an average of €2035 (95% CI 1937–2133). Predictors for total societal costs were retirement status, age, gender, alcohol intake, and back-related functioning. Retirement status emerged as the strongest predictor, leading us to divide the cohort into pensioners and non-pensioners to explore societal costs and relevant predictors for participants with a high-cost pattern.

The non-pensioners’ total societal costs mainly consisted of absenteeism and presenteeism costs (mean €7579 (95% CI 5877–9281), whereas the pensioners’ total societal costs were €1754 (95% CI 1010–2499). For the non-pensioners, gender, age, alcohol intake, and back-related functioning were predictors of being in the 20% highest cost category. In the Netherlands, males generally tend to have a higher income compared to women [31], which explains why they have higher absenteeism costs and therefore higher societal costs. Secondly, in this elderly population, having a younger age means that these people are still working, which is related to higher societal costs. The predictive value of alcohol intake is more difficult to understand. An explanatory factor might be that lower alcohol intake is related to healthier people, which is associated to less costs. This could, however, be a spurious finding, and would need to be corroborated by other more robust studies. Finally, as expected, participants with a worse back-related functioning have a higher likelihood to be in the high cost group. Interestingly, pain score was not related to the costs while they tend to follow the same pattern. A Pearson’s correlation test between pain and costs (healthcare and total costs) showed no correlation. This could potentially be explained by the fact that pain might be less relevant than functional status for someone’s ability to work.

All pensioners were in the lowest cost-category. It was impossible to predict high costs for the pensioners, due to low variability in their costs. Even when using the top 20% of highest costs within the group of non-pensioners, high costs could not be predicted for this group.

The model’s overall fit was good, with an explained variance (28.2%). The AUC was 0.86, indicating good interpretability. Internal validation had minimal effect on the model’s performance suggesting a low risk of overfitting.

### Comparison to the literature

Direct comparability of this study with other studies is limited, as few studies on costs related to LBP and, as far as we are aware, no similar study among older adults visiting chiropractic practices/study with a similar population and in a similar setting have been conducted in the Netherlands. Therefore, the Dutch results can be considered informative for other European countries, but a direct comparison is not possible [32–34].

The mean societal costs of this study were €5297 per patient for the first year after contacting a chiropractor, resembling a similar Dutch study by Mutubuki et al. (2020), reporting €5522 [5]. In the current study, participants were categorized as high-cost user (highest 20%) if their total annual societal costs were ≥ €8694. In the study of Mutubuki et al. (2020) [5] costs at different cut-off points were as follows: 20% (≥ €7906), 10% (≥ €11,922), and 5% (≥ €19,403).

Dutmer et al. (2019) [35] reported higher costs of €9000 per patient. This discrepancy is like explained by variations in healthcare system structures, patient settings and/or differences in cost estimations (administrative data versus self-reporting for example). Killingmo et al. [32] in a similar Norwegian cohort reported mean costs of €825 (682–976) per year per patient related to healthcare utilisation. Costs related to healthcare utilisation in this cohort (medication costs, primary and secondary healthcare) would add up to €1102 in one year, which is relatively comparable to the cohort of Killingmo. This is higher than the healthcare direct healthcare costs per patient for a recent Dutch study about general practitioner-guided care in patients with musculoskeletal complaints by Pellekooren et al. of €97 [32]. Even though the study of Pellekooren focused on a population similar to this study, their focus was on general practitioner-guided care costs only (instead of total societal costs) in patients with a broad range of musculoskeletal complains.

Few studies have investigated predictive factors for high societal costs among people with LBP. Gender, being male, found as a predictive factor for high societal costs in the current study, was also found in other studies [5, 34]. Other studies also found different predictive factors compared to those found in the current study, such as pain persistence, mental health issues, including high pain scores and comorbidities [6, 14, 15, 34–36]. A possible explanation for the differences found in predictive factors is that the studies took place in different healthcare settings in different countries with different insurance packages and different availability of primary and secondary healthcare. Comparing the outcome of this study with possible similar studies set in different countries is likely to be limited due to different ways of financial registration of healthcare costs in different countries as well [36].

### Strengths and limitations study

This study is the first to investigate societal costs and predictive factors in chiropractic patients aged 55 years or older with LBP in the Netherlands. Mapping societal costs and predictive factors is vital to decrease the use of scarce healthcare resources and reduce the burden on our healthcare systems [37]. The use of advanced methodology for handling missing data and performing prediction models enhances the study's reliability.

A limitation of this study is the possible underestimation of total healthcare utilisation and related costs due to using self-reported outcome measures. Self-reports tend to underestimate the true value of healthcare utilisation due to potential recall bias [38]. However, in the Netherlands administrative data for healthcare measures is practically inaccessible and for that reason we used self-reported data. On the other hand, presenteeism was self-reported, which might have resulted in an overestimation of these costs. That is, VAS-based presenteeism scales are prone to end-aversion bias [39], meaning, individuals to avoid extreme choices and select a choice in the middle of the scale. Consequently, participants might have underreported their productivity at work, which in turn led to overestimated presenteeism costs. Despite the above-stated limitations, this is the recommended method as there are no registers on healthcare-related costs available in the Netherlands, nor objective measures of work performance.

A second limitation is the potential overestimation of societal costs attributed to LBP for participants who have reported one or multiple comorbidities. Over half of the participants reported to have one or multiple comorbidities. These comorbidities might have contributed to their societal costs, while not directly relatable to their LBP, resulting in a potential overestimation of cost attributed to LBP. A recent Canadian study concluded that people identified as having chronic pain have a higher prevalence of comorbidities and use significantly more publicly funded health services [40].

A third limitation is the missing data, which is related to the use of self-reported outcomes. It is well-known that healthcare utilisation is prone to missing data and that missing values should be replaced to make use of all reported data. Multivariate imputation by chained equations has been used to handle the missing data, thereby avoiding complete-case analysis which would have significantly reduced the power of these findings and potentially introduced information bias due to selective drop-out of participants [27]. This is the preferred statistical method for dealing with missing data, particularly when costs are involved [41, 42].

Another limitation is the small sample size. The focus on people who visited the chiropractor led to a selection of a relatively narrow group of participants. However, we consider this a strength of this study as information on predictors for costs in this population is lacking, whereas exploring the mechanisms related to high-cost users could potentially lead to implementation initiatives or modification of policy aimed at reducing costs. The selection of patients should be considered when generalizing our results. Secondly, the limited sample size limited us in exploring different cut-off point for high costs. Secondly, using more explanatory trajectory analyses (e.g. sequence analysis) were not possible. As this is an exploratory study and the first study ever done evaluating the costs in this population, the outcome is valuable as an indication for further research. Despite these limitations, the study provides valuable insights and serves as a foundation for future research.

### Implications for research and practice

This study recommends separating pensioners from non-pensioners in future research, given the differences in cost patterns and predictors. Establishing a consensus on cut-off points for high costs would enhance comparability with existing literature. Understanding the mechanisms associated with identified predictors for high societal costs is crucial for facilitating cost reductions.

This is an exploratory study, of which the importance is to emphasize the need to study older adults with chronic LBP. While this study will not have direct implications for daily clinical practice, it could influence guideline commissions who are confronted with costs for people with LBP who continue to work or are retired to offer targeted interventions to prevent, a decline in health status and subsequent influence on their work status and substantial avoidable costs.

## Conclusion

This study estimated the mean annual societal cost to be €5297 (95% CI 4191–6403). Most costs were made in the first 3 months, slightly declined, and remained stable for the rest of the year. "Working" emerged as the main predictor of high costs in this population of older adults. Future studies focusing on older adults should explore pensioners and non-pensioners separately, delving into the mechanisms associated with identified predictors for high societal costs to facilitate effective cost reductions.

## Supplementary Information

Additional file 1.

Additional file 2.

Additional file 3.

Additional file 4.

Additional file 5.

## Data Availability

The datasets used and/or analysed during the current study are available from the corresponding author on reasonable request.

## Abbreviations

BACE-C: The back complaints in elderly-chiropractic study
LBP: Low back pain
95% CI: 95% Confidence interval
OR: Odds ratio
BMI: Body Mass Index
QALY: Quality adjusted life years
RMDQ: Roland Morrison Disability Index
SD: Standard deviation
AUC: Area under the receiver operating characteristics curve
NRS: Numeric Rating Scale
GPE: Global perceived effect

## References

[1] Vos T, Lim SS, Abbafati C, Abbas KM, Abbasi M, Abbasifard M, et al. Global burden of 369 diseases and injuries in 204 countries and territories, 1990–2019: a systematic analysis for the Global Burden of Disease Study 2019. The Lancet. 2020;396(10258):1204–22. 10.1016/S0140-6736(20)30925-9 PMC7567026 33069326

[2] Hartvigsen J, Hancock MJ, Kongsted A, Louw Q, Ferreira ML, Genevay S, et al. What low back pain is and why we need to pay attention. The Lancet. 2018;391(10137):2356–67. 10.1016/S0140-6736(18)30480-X 29573870

[3] Atella V, Piano Mortari A, Kopinska J, Belotti F, Lapi F, Cricelli C, et al. Trends in age-related disease burden and healthcare utilization. Aging Cell. 2019;18(1): e12861. 30488641 10.1111/acel.12861 PMC6351821

[4] Fund TC. The performance improvement imperative: utilizing a coordinated, community-based approach to enhance care and lower costs for chronically Ill patients; 2012.

[5] Mutubuki EN, Luitjens MA, Maas ET, Huygen FJPM, Ostelo RWJG, van Tulder MW, et al. Predictive factors of high societal costs among chronic low back pain patients. Eur J Pain. 2020;24(2):325–37. 31566839 10.1002/ejp.1488 PMC7003839

[6] Foster NE, Anema JR, Cherkin D, Chou R, Cohen SP, Gross DP, et al. Prevention and treatment of low back pain: evidence, challenges, and promising directions. (1474–547X (Electronic)). 10.1016/S0140-6736(18)30489-6 29573872

[7] Schnitzler LRT, Jackson LJ, Paulus ATG, Evers SMAA. A consensus-based checklist for the critical appraisal of cost-of-illness (COI) studies. Int J Technol Assess Health Care. 2023;39(1):e34. 37325977 10.1017/S0266462323000193 PMC11574538

[8] Jenks AD, Hoekstra T, Axén I, De Luca K, Field J, Newell D, et al. BAck complaints in the elders-chiropractic (BACE-C): protocol of an international cohort study of older adults with low back pain seeking chiropractic care. Chiropract Manual Ther. 2020;28(1):1–7. 10.1186/s12998-020-00302-z PMC7110664 32238185

[9] Scheele J, Luijsterburg PAJ, Ferreira ML, Maher CG, Pereira L, Peul WC, et al. Back complaints in the elders (BACE); design of cohort studies in primary care: an international consortium. BMC Musculoskelet Disord. 2011;12(1):1–9. 21854620 10.1186/1471-2474-12-193 PMC3182961

[10] Goossens MEJB, Rutten-van Mölken MPMH, Vlaeyen JWS, van der Linden SMJP. The cost diary: a method to measure direct and indirect costs in cost-effectiveness research. J Clin Epidemiol. 2000;53(7):688–95. 10941945 10.1016/s0895-4356(99)00177-8

[11] Kanters TA, Bouwmans CAM, Van Der Linden N, Tan SS, Hakkaart-van RL. Update of the Dutch manual for costing studies in health care. PLoS ONE. 2017;12(11): e0187477. 29121647 10.1371/journal.pone.0187477 PMC5679627

[12] Hakkaart-van Roijen L, Van der Linden N, Bouwmans C, Kanters T, Tan SS. Kostenhandleiding. Methodologie van kostenonderzoek en referentieprijzen voor economische evaluaties in de gezondheidszorg In opdracht van Zorginstituut Nederland Geactualiseerde versie. 2015;12–64.

[13] Koopmanschap MA. PRODISQ: a modular questionnaire on productivity and disease for economic evaluation studies. Expert Rev Pharmacoecon Outcomes Res. 2005;5(1):23–8. 19807557 10.1586/14737167.5.1.23

[14] Koopmanschap MA, Rutten FFH, Van Ineveld BM, Van Roijen L. The friction cost method for measuring indirect costs of disease. J Health Econ. 1995;14(2):171–89. 10154656 10.1016/0167-6296(94)00044-5

[15] Becker A, Held H, Redaelli M, Strauch K, Chenot JF, Leonhardt C, et al. Low back pain in primary care: costs of care and prediction of future health care utilization. Spine. 2010;35(18):1714–20. 21374895 10.1097/brs.0b013e3181cd656f

[16] Engel CC, Von Korff M, Katon WJ. Back pain in primary care: predictors of high health-care costs. Pain. 1996;65(2–3):197–204. 8826507 10.1016/0304-3959(95)00164-6

[17] Berk ML, Monheit AC. The concentration of health care expenditures, revisited. Health Aff. 2001;20(2):9–18. 10.1377/hlthaff.20.2.9 11260963

[18] Chechulin Y, Nazerian A, Rais S, Malikov K. Predicting patients with high risk of becoming high-cost healthcare users in Ontario (Canada). Healthc Policy. 2014;9(3):68. 24726075 PMC3999564

[19] Childs JD, Piva SR, Fritz JM. Responsiveness of the numeric pain rating scale in patients with low back pain. Spine. 2005;30(11):1331–4. 15928561 10.1097/01.brs.0000164099.92112.29

[20] Roland M, Fairbank J. The Roland–Morris disability questionnaire and the Oswestry disability questionnaire. Spine. 2000;25(24):3115–24. 11124727 10.1097/00007632-200012150-00006

[21] Devilly GJ, Borkovec TD. Psychometric properties of the credibility/expectancy questionnaire. J Behav Ther Exp Psychiatry. 2000;31(2):73–86. 11132119 10.1016/s0005-7916(00)00012-4

[22] Frank D, DeBenedetti AF, Volk RJ, Williams EC, Kivlahan DR, Bradley KA. Effectiveness of the AUDIT-C as a screening test for alcohol misuse in three race/ethnic groups. J Gen Intern Med. 2008;23:781–7. 18421511 10.1007/s11606-008-0594-0 PMC2517893

[23] Buysse DJ, Reynolds Iii CF, Monk TH, Berman SR, Kupfer DJ. The pittsburgh sleep quality index: a new instrument for psychiatric practice and research. Psychiatry Res. 1989;28(2):193–213. 2748771 10.1016/0165-1781(89)90047-4

[24] Sangha O, Stucki G, Liang MH, Fossel AH, Katz JN. The self-administered comorbidity Questionnaire: a new method to assess comorbidity for clinical and health services research. Arthr Care Rese Off J Am College of Rheumatol. 2003;49(2):156–63. 10.1002/art.10993 12687505

[25] Bier JD, Ostelo RWJG, Van Hooff ML, Koes BW, Verhagen AP. Validity and reproducibility of the start back tool (Dutch version) in patients with low back pain in primary care settings. Phys Ther. 2017;97(5):561–70. 28340202 10.1093/ptj/pzx023

[26] Whynes DK, McCahon RA, Ravenscroft A, Hodgkinson V, Evley R, Hardman JG. Responsiveness of the EQ-5D health-related quality-of-life instrument in assessing low back pain. Value Health. 2013;16(1):124–32. 23337223 10.1016/j.jval.2012.09.003

[27] White IR, Royston P, Wood AM. Multiple imputation using chained equations: issues and guidance for practice. Stat Med. 2011;30(4):377–99. 21225900 10.1002/sim.4067

[28] Harrell FE Jr, Lee KL, Mark DB. Multivariable prognostic models: issues in developing models, evaluating assumptions and adequacy, and measuring and reducing errors. Stat Med. 1996;15(4):361–87. 8668867 10.1002/(SICI)1097-0258(19960229)15:4<361::AID-SIM168>3.0.CO;2-4

[29] Steyerberg EW, Vickers AJ, Cook NR, Gerds T, Gonen M, Obuchowski N, et al. Assessing the performance of prediction models: a framework for some traditional and novel measures. Epidemiology. 2010;21(1):128. 20010215 10.1097/EDE.0b013e3181c30fb2 PMC3575184

[30] Tsuboi Y, Murata S, Naruse F, Ono R. Association between pain-related fear and presenteeism among eldercare workers with low back pain. Eur J Pain. 2019;23(3):495–502. 30289190 10.1002/ejp.1323

[31] Statistiek CBvd. Loonverschil tussen mannen en vrouwen in 2022 iets afgenomen 2023. https://www.cbs.nl/nl-nl/nieuws/2023/49/loonverschil-tussen-mannen-en-vrouwen-in-2022-iets-afgenomen#:~:text=In%202022%20verdienden%20vrouwen%20gemiddeld,en%2023%2C30%20euro

[32] Killingmo RM, Storheim K, van der Windt D, Zolic-Karlsson Z, Vigdal ØN, Kretz L, et al. Healthcare utilization and related costs among older people seeking primary care due to back pain: findings from the BACE-N cohort study. BMJ Open. 2022;12(6): e057778. 35725262 10.1136/bmjopen-2021-057778 PMC9214384

[33] Wenig CM, Schmidt CO, Kohlmann T, Schweikert B. Costs of back pain in Germany. Eur J Pain. 2009;13(3):280–6. 18524652 10.1016/j.ejpain.2008.04.005

[34] Wieser S, Horisberger B, Schmidhauser S, Eisenring C, Brügger U, Ruckstuhl A, et al. Cost of low back pain in Switzerland in 2005. Eur J Health Econ. 2011;12:455–67. 20526649 10.1007/s10198-010-0258-y PMC3160551

[35] Dutmer AL, Preuper HRS, Soer R, Brouwer S, Bültmann U, Dijkstra PU, et al. Personal and societal impact of low back pain: the Groningen spine cohort. Spine. 2019;44(24):E1443–51. 31369481 10.1097/BRS.0000000000003174

[36] Itz CJ, Ramaekers BLT, Van Kleef M, Dirksen CD. Medical specialists care and hospital costs for low back pain in the Netherlands. Eur J Pain. 2017;21(4):705–15. 27860026 10.1002/ejp.974

[37] Buchbinder R, van Tulder M, Öberg B, Costa LM, Woolf A, Schoene M, et al. Low back pain: a call for action. The Lancet. 2018;391(10137):2384–8. 10.1016/S0140-6736(18)30488-4 29573871

[38] Icks A, Dittrich A, Brüne M, Kuss O, Hoyer A, Haastert B, et al. Agreement found between self-reported and health insurance data on physician visits comparing different recall lengths. J Clin Epidemiol. 2017;82:167–72. 27825891 10.1016/j.jclinepi.2016.10.009

[39] Gay KD, Leal A, Ruth TK, Lamm AJ, Rumble JN. Comparing the use of visual analogue scales and likert-type scales in international agricultural and extension education surveys. J Int Agric Extens Educ. 2015;22(2):37–51.

[40] Foley HE, Knight JC, Ploughman M, Asghari S, Audas R. Association of chronic pain with comorbidities and health care utilization: a retrospective cohort study using health administrative data. Pain. 2021;162(11):2737–49. 33902092 10.1097/j.pain.0000000000002264

[41] Ben ÂJ, van Dongen JM, Alili ME, Heymans MW, Twisk JWR, MacNeil-Vroomen JL, et al. The handling of missing data in trial-based economic evaluations: should data be multiply imputed prior to longitudinal linear mixed-model analyses? Eur J Health Econ. 2023;24(6):951–65. 36161553 10.1007/s10198-022-01525-y PMC10290620

[42] El Alili M, van Dongen JM, Esser JL, Heymans MW, van Tulder MW, Bosmans JE. A scoping review of statistical methods for trial-based economic evaluations: The current state of play. Health Econ. 2022;31(12):2680–99. 36089775 10.1002/hec.4603 PMC9826466

