# Supplementary material for: Societal costs of older adults with low back pain seeking chiropractic care: findings from the BACE-C cohort study
Source: Chiropr Man Therap. 2024 Nov 6;32:31. doi: 10.1186/s12998-024-00553-0 (PMC11539272; doi:10.1186/s12998-024-00553-0)
Supplement: Supplementary file 1 — Additional file 1. [file 12998_2024_553_MOESM1_ESM.docx]

| **Checklist for cost-of-illness studies: Societal costs BACE-C study** | | | |  |  |  |
| --- | --- | --- | --- | --- | --- | --- |
| **Item** | **Question** | **Answer** | **Supportive information** | | | |
| **Study characteristics** |  |  |  | | | |
| Question/objective | 1) Is a well-defined research question or objective stated? | Yes | 2 objectives are described | | | |
| Population | 2) Is the study population described? | Yes | The study population is described under the caption ‘Participants’ | | | |
| Perspective | 3a) Is (are) the chosen study perspective(s) stated? | Yes | This is described under ‘study design and setting’ | | | |
|  | 3b) If so, is (are) the chosen study perspective(s) justified? | Yes | This is described in 3rd Alinea introduction | | | |
| **Methodology and cost analysis** |  |  |  | | | |
| Epidemiological approach | 4) Is the epidemiological approach reported (e.g. prevalence, incidence)? | Yes | Methods – First paragraph | | | |
| Costing approach | 5) Is the costing approach reported (e.g., top-down, bottom-up)? | Yes | The cost approach is reported under the caption outcome measures | | | |
| Data-collection approach | 6) Is the data collection process reported (e.g., prospective, retrospective)? | Yes | This is reported under the caption ‘Study design and setting’ | | | |
| Identification | 7a) Are all components of resource use identified that are relevant to the condition/disease, population, intervention, study objectives, and study perspective? | Yes | These are described under ‘Outcome measures’ and ‘Potential predictive factors’ | | | |
|  | 7b) If not, is a justification provided for excluding relevant components of resource use? | NA |  | | | |
| Measurement | 8a) Are all included components of resource use measured? | Yes | These are described under the captions ‘Outcome measures’ and ‘Potential predictive factors’. | | | |
|  | 8b) If not, is a justification provided for not measuring certain components of resource use? | NA |  | | | |
| Valuation | 9a) Are all included components of resource use valued in monetary terms? | Yes | This is described under the caption ‘Statistical analyses’ | | | |
|  | 9b) If not, is a justification provided for not valuing certain components of resource use? | NA |  | | | |
| Time horizon | 10a) Is the chosen time horizon specified? | Yes | This is described under ‘Statistical Analyses’ | | | |
|  | 10b) If so, is the chosen time horizon justified? | Yes | This justification is described under ‘Study Design and setting’ and ‘Outcome measures’ | | | |
| Discounting | 11a) Are future costs discounted? | NA |  | | | |
|  | 11b) If so, is a justification provided for the discount rate? | NA |  | | | |
| Sensitivity | 12a) Are all variables whose values are uncertain subjected to sensitivity analysis? | Yes | This is described under ‘Statistical Analyses | | | |
|  | 12b) If so, is a justification provided for which variables are subjected to sensitivity analysis? | Yes |  | | | |
|  | 12c) Are analyses done on relevant subgroups? | Yes | This is described under the caption ‘Subgroup analyses’ | | | |
| **Results and reporting** |  |  |  | | | |
| Cost sectors | 13) Are the study results presented transparently by cost category/sector? | Yes | E.g. Table 2 | | | |
| Generalisability | 14) Do the authors discuss the generalisability of study results (e.g., comparing the results to other patient/client groups or/in other settings? | Yes | This is described under the caption “Comparison to the literature’ | | | |
| Limitations | 15) Do the authors discuss important limitations? | Yes | Important limitations are discussed under the caption ‘Strengths and limitations study’ | | | |
| Ethical and distributional issues | 16a) Do the authors discuss ethical issues? | NA |  | | | |
|  | 16b) Do the authors discuss distributional issues? | Yes | This is discussed under the caption ‘Main findings’ as well as under the caption ‘Implication for research and practice’ | | | |
| Conflict of interest | 17) Do the authors report any potential conflicts of interest? | No | The authors report there is no conflict of interest | | | |
