# Supplementary material for: Societal costs of older adults with low back pain seeking chiropractic care: findings from the BACE-C cohort study
Source: Chiropr Man Therap. 2024 Nov 6;32:31. doi: 10.1186/s12998-024-00553-0 (PMC11539272; doi:10.1186/s12998-024-00553-0)
Supplement: Supplementary file 2 — Additional file 2. [file 12998_2024_553_MOESM2_ESM.pdf]

BACK PAIN THE ELDERLY & CHIROPRACTIC CARE

BASELINE QUESTIONNAIRE

Patient Code :

AGE, GENDER, ETHNICITY, EDUCATION, MARITAL STATUS, HEIGHT, WEIGHT

**Today's Date:**	Click here to enter a date.		
**First Name:**	Enter First Name	**Last Name:**	Enter Last Name
**Address:**	Enter here	**Postal Code:**	Enter here
**City:**	Enter here	**eMail:**	Enter here
**Mobile #:**		**Birthdate:**	Enter here
**Height:**		**Weight:**	

1) What is your gender?
   - ☐ Male
   - ☐ Female
   - ☐ Prefer not to say
2) What is your marital status?
   - ☐ Single
   - ☐ Married or Living together
   - ☐ Living apart together
3) In which country were you born?
   - ☐ Netherlands
   - ☐ Sweden
   - ☐ United Kingdom
   - ☐ Other, namely………………….
4) In which country was your mother born?
   - ☐ Netherlands
   - ☐ Sweden
   - ☐ United Kingdom
   - ☐ Other, namely……………….
5) In which country was your father born?
   - ☐ Netherlands
   - ☐ Sweden
   - ☐ United Kingdom
   - ☐ Other, namely………………….

6) What is the highest level of education that you have achieved? (Use equivalent Swedish & English school levels)
   - ☐ Geen
   - ☐ Lagere school
   - ☐ Lager beroepsonderwijs (huishoudschool, LTS, LEAO, LBE, enz.)
   - ☐ Middelbaar algemeen voortgezet onderwijs (MAVO, IVO, (M)ULO, VMBO, MMS)
   - ☐ Hoger algemeen, voorbereidend wetenschappelijk onderwijs (HAVO, VWO, HBS, MMS, Gymnasium, Atheneum)
   - ☐ Middelbaar beroepsonderwijs (MTS, MEAO, MHNO, BOL, INAS, enz.)
   - ☐ Hoger beropsonderwijs (HBO, HTS, HEAO, Sociale Academie, enz.)
7) What do you do? Place an x in the box for what you usually do.
   - ☐ I go to school, I am studying
   - ☐ I am employed
   - ☐ I am self-employed
   - ☐ I am a stay-at-home women/man
   - ☐ I am unemployed
   - ☐ I am unable to work for ……….. %
   - ☐ I am retired or on a pre-pension plan
   - ☐ I do something else, namely ……………………

SEVERITY, REOCCURANCE, DURATION, ONSET, FREQUENCY, RADIATION, NUMBNESS, WEAKNESS

8) Do you know how the low back pain started?
   - ☐ Suddenly with a bad movement
   - ☐ Suddenly from heavy lifting
   - ☐ Accident/trauma to the low back
   - ☐ Slowly over days
   - ☐ Other…………
9) How often do you have low back pain?
   - ☐ less than 1 times per week

☐ 1 times per week

☐ Every day for a few minutes

☐ Every day, for most of the day

☐ Every minute of every day

10) What area(s) do you feel most of the low back pain? (more than one box allowed)

☐ Low back

☐ Leg

☐ Neck

☐ Headache

☐ Shoulder/arm

☐ Other, ……………………………………………….

11) Have you had this **SAME** or a similar low back pain anytime in the **PAST**?

☐ Yes

☐ No

12) Have you had a **WHOLE MONTH** in the **past 6 months WITHOUT** pain from a similar complaint?

☐ Yes

☐ No

13) How long has this **PRESENT EPISODE** of your low back pain lasted?

☐☐ days/weeks/months/years

14) How would you describe this **PRESENT EPISODE** of your pain?

☐ Comes and goes

☐ There constantly

15) Has this **PRESENT EPISODE** of your painful complaint been bad enough to limit your usual activities or change your daily routine for **MORE THAN ONE DAY**?

☐ Yes

☐ No

16) On a scale of 0 (no pain) to 10 (worst pain imaginable). Select the number the best represents your **low back pain** at this moment.

No										Worst Pain

Pain										Imaginable
☐	☐	☐	☐	☐	☐	☐	☐	☐	☐	☐
0	1	2	3	4	5	6	7	8	9	10

17) How bad was the **low back pain** <u>in the last week</u>?

No Pain										Worst Pain Imaginable
☐	☐	☐	☐	☐	☐	☐	☐	☐	☐	☐
0	1	2	3	4	5	6	7	8	9	10

18) Does your low back pain refer pain to your **leg**?

- ☐ No, **go to question ##**
- ☐ Buttocks
- ☐ Hips
- ☐ Yes, to the upper leg
- ☐ Yes, to the lower leg
- ☐ Yes, to the foot

19) Where do you feel the pain in your leg?

- ☐ Left
- ☐ Right
- ☐ Both legs

20) How bad is the pain in your **leg** <u>at this moment</u>?

No Pain										Worst Pain Imaginable
☐	☐	☐	☐	☐	☐	☐	☐	☐	☐	☐
0	1	2	3	4	5	6	7	8	9	10

21) How bad was the pain in the **leg** <u>in the last week</u>?

No Pain										Worst Pain Imaginable
☐	☐	☐	☐	☐	☐	☐	☐	☐	☐	☐
0	1	2	3	4	5	6	7	8	9	10

22) To what extent have you in the past week had a numbness or tingling in your leg or foot?

None	Mild	Moderate	Serious	Very Serious
☐	☐	☐	☐	☐

23) To what extent have you in the last week a weak or a heavy feeling in your leg or foot?

None	Mild	Moderate	Serious	Very Serious
☐	☐	☐	☐	☐

24) How far were you able to on an average day maximally walk in the last week?

More than 3 Km	200 m -3 Km	15 m – 200 m	Less than 15 m
☐	☐	☐	☐

25) How do you expect your condition to RESPOND TO TREATMENT?

☐ Recover/Improve

☐ Stay about the same

☐ Get worse

26) I am worried about my treatment.

☐ Yes

☐ No

THE ROLAND-MORRIS DISABILITY QUESTIONNAIRE

When your back hurts, you may find it difficult to do some of the things you normally do.

This list contains sentences that people have used to describe themselves when they have back pain. When you read them, you may find that some stand out because they describe you *today*.

As you read the list, think of yourself *today*. When you read a sentence that describes you today, put a tick against it. If the sentence does not describe you, then leave the space blank and go on to the next one. Remember, only tick the sentence if you are sure it describes you today.

- ☐ I stay at home most of the time because of my back.
- ☐ I change position frequently to try and get my back comfortable.
- ☐ I walk more slowly than usual because of my back.
- ☐ Because of my back I am not doing any of the jobs that I usually do around the house.
- ☐ Because of my back, I use a handrail to get upstairs.
- ☐ Because of my back, I lie down to rest more often.
- ☐ Because of my back, I have to hold on to something to get out of an easy chair.
- ☐ Because of my back, I try to get other people to do things for me.
- ☐ I get dressed more slowly than usual because of my back.
- ☐ I only stand for short periods of time because of my back.
- ☐ Because of my back, I try not to bend or kneel down.
- ☐ I find it difficult to get out of a chair because of my back.
- ☐ My back is painful almost all the time.
- ☐ I find it difficult to turn over in bed because of my back.
- ☐ My appetite is not very good because of my back pain.
- ☐ I have trouble putting on my socks (or stockings) because of the pain in my back.
- ☐ I only walk short distances because of my back.
- ☐ I sleep less well because of my back.
- ☐ Because of my back pain, I get dressed with help from someone else.
- ☐ I sit down for most of the day because of my back.
- ☐ I avoid heavy jobs around the house because of my back.
- ☐ Because of my back pain, I am more irritable and bad tempered with people than usual.
- ☐ Because of my back, I go upstairs more slowly than usual.
- ☐ I stay in bed most of the time because of my back

Reoccurrence of Back Pain:

13. Have you had a WHOLE MONTH in the past 6 months WITHOUT any pain from a similar complaint?
    - ☐ Yes
    - ☐ No
14. How would you describe this PRESENT EPISODE of your pain?
    - ☐ Comes and goes
    - ☐ There constantly
15. Has this PRESENT EPISODE of your painful complaint been bad enough to limit your usual activities or change your daily routine for MORE THAN ONE DAY?
    - ☐ Yes
    - ☐ No

EQ-5D-5L:

Under each heading, please tick the ONE box that best describes your health TODAY

MOBILITY

- ☐ I have no problems in walking about
- ☐ I have slight problems in walking about
- ☐ I have moderate problems in walking about
- ☐ I have severe problems in walking about
- ☐ I am unable to walk about

SELF-CARE

- ☐ I have no problems washing or dressing myself
- ☐ I have slight problems washing or dressing myself
- ☐ I have moderate problems washing or dressing myself
- ☐ I have severe problems washing or dressing myself
- ☐ I am unable to wash or dress myself

USUAL ACTIVITIES *(e.g. work, study, housework, family or leisure activities)*

- ☐ I have no problems doing my usual activities
- ☐ I have slight problems doing my usual activities
- ☐ I have moderate problems doing my usual activities
- ☐ I have severe problems doing my usual activities
- ☐ I am unable to do my usual activities

PAIN / DISCOMFORT

- ☐ I have no pain or discomfort
- ☐ I have slight pain or discomfort
- ☐ I have moderate pain or discomfort
- ☐ I have severe pain or discomfort
- ☐ I have extreme pain or discomfort

ANXIETY / DEPRESSION

- ☐ I am not anxious or depressed
- ☐ I am slightly anxious or depressed
- ☐ I am moderately anxious or depressed
- ☐ I am severely anxious or depressed
- ☐ I am extremely anxious or depressed

We would like to know how good or bad your health is TODAY.

- This scale is numbered from 0 to 100.
-
- 100 means the best health you can imagine.

- 0 means the worst health you can imagine.

- Mark an X on the scale to indicate how your health is TODAY.

- Now, please write the number you marked on the scale in the box below.

YOUR HEALTH TODAY =

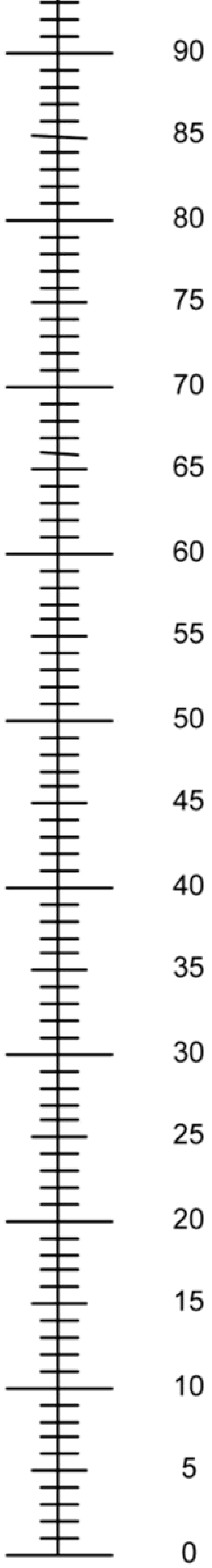

The worst health
you can imagine

COST EFFECTIVENESS QUESTIONNAIRE

Part 1 – Healthcare utilization

1. During the <u>past three months</u>, ...?
   - [illegible]
   - did you visit an emergency department, <u>or</u>
   - have you been submitted to a hospital, <u>or</u>
   - did you use prescription drugs

☐ No → go to question 10
☐ Yes → go to question 2

2. Did you consult a general practitioner during the **<u>past three months</u>**?

☐ No → go to question 3
☐ Yes → please fill out the following Table

**Type of consultation**	**Number of consultations**
Practice visit	…………
Telephone consultation	…………
House call	…………

3. **Did you visit an allied health professional during the <u>past three months</u>?**

☐ No → go to question 4
☐ Yes → please fill out the following Table

**Allied health professional**	**Number of visits**
Physical therapist	………
Occupational therapist	………
Ceasar or Mensendieck therapist	………
Chiropractor	………

Manual therapist	………
Psychologist	………
Other, ……………………………………	………
Other, ……………………………………	………

4. **Did you visit a complementary care provider during the <u>past three months</u>? (For the low back pain ?)**

☐ No → go to question 5
☐ Yes → please fill out the following Table

**Complementary care provider**	**Cost per visit**	**Number of visits**
Acupuncturist	€ ………	………
Homeopath	€ ………	………
Holistic therapist	€ ………	………
Natural healer	€ ………	………
Other, ……………………………………	€ ………	………
Other, ……………………………………	€ ………	………

5. **Did you visit an emergency department during the <u>past three months</u>?**

☐ No → go to question 6
☐ Yes → please fill out the following Table

**Number of visits**	**Were you brought to the emergency department by ambulance?**
…………	☐ No<br>☐ Yes, ……… (number of rides)

6. **Did you visit a medical specialist of an outpatient clinic during the <u>past three months</u>?**

☐ No → go to question 7
☐ Yes → please fill out the following Table

Medical specialist	Number of visits
Cardiologist	………
Orthopedic surgeon	………
Neurosurgeon	………
Rehabilitation physician	………
Psychiatrist	………
Other, ……………………	………
Other, ……………………	………

7. Have you in the last 3 months for your low back pain had a:

Bloodtest

☐ Yes
☐ No

Xrays of your back

☐ Yes
☐ NO

MRI/CT-scan of your back

☐ Yes
☐ No

8. Have you been admitted to a hospital during the <u>past three months</u>?

☐ No → go to question 8
☐ Yes → please fill out the following Table

**Hospital admission**	**Date of admission** *(dd/mm/yyyy)*	**Date of discharge** *(dd/mm/yyyy)*	**Were you admitted to an intensive care unit (ICU)?**

**Admission 1**	**..../ /......**	**..../ /......**	☐ No<br>☐ Yes, for .... days
**Admission 2**	**..../ /......**	**..../ /......**	☐ No<br>☐ Yes, for .... days

9. Did you visit other kinds of healthcare providers during the <u>past three months?</u>

☐ No → go to question 9
☐ Yes → please fill out the following Table

**Kind of healthcare providers**	**Number of visits**
……………………………………………………………………………<br>……………………………………………………………………………<br>……………………………………………………………………………	………<br>………<br>………

10. Did you use prescription drugs during the <u>past three months</u>? Examples of prescription drugs are diclofenac, citalopram, tramadol. (pain prescription drugs or just prescription drugs)

☐ No → go to question 10
☐ Yes → please fill out the following Table

**Kind of prescription drugs**	**Dosage form**	**Daily dose (as indicated on the label)**	**For how many days did you use the drugs during the <u>past three months</u>?**

**Name (1):**<br><br>____________________	**Tablet / capsule**<br>**Cream / ointment**<br>**Inhaler**<br>**Other, ……**	....	.... days
**Name (2):**<br><br>____________________	**Tablet / capsule**<br>**Cream / ointment**<br>**Inhaler**<br>**Other, ……**	....	.... days
**Name (3):**<br><br>____________________	**Tablet / capsule**<br>**Cream / ointment**<br>**Inhaler**<br>**Other, ……**	....	.... days
**Name (4):**<br><br>____________________	**Tablet / capsule**<br>**Cream / ointment**<br>**Inhaler**<br>**Other, ……**	....	.... days
**Name (5):**<br><br>____________________	**Tablet / capsule**<br>**Cream / ointment**<br>**Inhaler**<br>**Other, ……**	....	.... days

INTERNATIONAL PHYSICAL ACTIVITY QUESTIONNAIRE (IPAQ-SHORT)

We are interested in finding out about the kinds of physical activities that people do as part of their everyday lives. The questions will ask you about the time you spent being physically active in the last 7 days. Please answer each question even if you do not consider yourself to be an active person. Please think about the activities you do at work, as part of your house and yard work, to get from place to place, and in your spare time for recreation, exercise or sport.

Think about all the vigorous activities that you did in the last 7 days. Vigorous physical activities refer to activities that take hard physical effort and make you breathe much harder than normal. Think only about those physical activities that you did for at least 10 minutes at a time.

11. During the last 7 days, on how many days and how much time did you do **vigorous** physical activities like heavy lifting, digging, aerobics, or fast bicycling?

☐ days per week? ☐ hours per day ☐ minutes per day ☐ Don’t know/Not sure

Think about all the moderate activities that you did in the last 7 days. Moderate activities refer to activities that take moderate physical effort and make you breathe somewhat harder than normal. Think only about those physical activities that you did for at least 10 minutes at a time.

12. During the last 7 days, on how many days and how much time did you do moderate physical activities like carrying light loads, bicycling at a regular pace, or doubles tennis? Do not include walking.

☐ days per week? ☐ hours per day ☐ minutes per day ☐ Don’t know/Not sure

Think about the time you spent walking in the last 7 days. This includes at work and at home, walking to travel from place to place, and any other walking that you have done solely for recreation, sport, exercise, or leisure.

13. During the last 7 days, on how many days and how much time did you walk for at least 10 minutes at a time?

☐ days per week? ☐ hours per day ☐ minutes per day ☐ Don’t know/Not sure

The last question is about the time you spent sitting on weekdays during the last 7 days. Include time spent at work, at home, while doing course work and during leisure time. This may include time spent sitting at a desk, visiting friends, reading, or sitting or lying down to watch television.

14. During the last 7 days, how much time did you spend sitting on a week day?

days per week? hours per day minutes per day Don’t know/Not sure

Sleeping

The following questions deal with your most recent sleeping problems in the last month. Try to answer what things are true that apply in the last month.

15. How often in the last month have you slept poorly due to your low back pain
    - ☐ Never
    - ☐ Less than 1-2x per week
    - ☐ 3x per week or more
16. How would you describe the quality of your sleep in the last month.
    - ☐ Very Good
    - ☐ Almost Good
    - ☐ Almost Bad
    - ☐ Very Bad

Physical Activity

17. Compared with people of a similar age and in a similar position, how would you rate your OVERALL PHYSICAL ACTIVITY?
    - ☐ More
    - ☐ About the same
    - ☐ Less

Comorbidity Questionnaire (SCQ)

Instructions: The following is a list of common health problems. Please indicate (circle correct answer) if you currently have the problem in the first column. If you do not have the problem, skip to the

next problem. If you do have the problem, please indicate in the second column if you receive medications or some other type of treatment for the problem. Also, indicate in that case in the third column if the problem limits any of your activities. Finally, indicate also medical conditions that are not listed under "other medical problems" at the end of the list.

	Do you have the problem?		Do you receive treatment for it?		Does it limit your activities	
Problem	Yes	No	Yes	No	Yes	No
Heart disease	Y	N	Y	N	Y	N
High blood pressure	Y	N	Y	N	Y	N
Lung disease	Y	N	Y	N	Y	N

Diabetes	Y	N	Y	N	Y	N	
Ulcer or stomach disease	Y	N	Y	N	Y	N	
Kidney disease	Y	N	Y	N	Y	N	
Liver disease	Y	N	Y	N	Y	N	
Anemia or other blood disease	Y	N	Y	N	Y	N	
Cancer	Y	N	Y	N	Y	N	
Depression	Y	N	Y	N	Y	N	
Pain and swelling in joints other than back	Y	N	Y	N	Y	N	
Osteoposoris	Y	N	Y	N	Y	N	
Fractures	Y	N	Y	N	Y	N	
	Y	N	Y	N	Y	N	
Other medical problems (please write in):							
1.	Y	N	Y	N	Y	N	
2.	Y	N	Y	N	Y	N	
3.	Y	N	Y	N	Y	N	

	**Agree**	**Disagree**
My back pain has **spread down my leg(s)** at some time in the last 2 weeks		
I have had pain in the **shoulder** or **neck** at some time in the last 2 weeks		
I have only **walked short distances** because of my back pain		
In the last 2 weeks, I have **dressed more slowly** than usual because of back pain		
It’s not really safe for a person with a condition like mine to be physically active		
**Worrying thoughts** have been going through my mind a lot of the time		
I feel that **my back pain is terrible** and **it’s never going to get any better**		
In general I have **not enjoyed** all the things I used to enjoy		

Thinking about the **last 2 weeks** tick your response to the following questions:

Overall, how **bothersome** has your back pain been in the **last 2 weeks**?

Not at all	Slightly	Moderately	Quite a bit	Extremely
☐	☐	☐	☐	☐

START BACK

Thinking about the **last 2 weeks** tick your response to the following questions:

		Disagree 0	Agree 1
1	My back pain has **spread down my leg(s)** at some time in the last 2 weeks	☐	☐
2	I have had pain in the **shoulder** or **neck** at some time in the last 2 weeks	☐	☐
3	I have only **walked short distances** because of my back pain	☐	☐
4	In the last 2 weeks, I have **dressed more slowly** than usual because of back pain	☐	☐
5	It's not really safe for a person with a condition like mine to be physically active	☐	☐
6	**Worrying thoughts** have been going through my mind a lot of the time	☐	☐
7	I feel that **my back pain is terrible** and **it's never going to get any better**	☐	☐
8	In general I have **not enjoyed** all the things I used to enjoy	☐	☐

9. Overall, how **bothersome** has your back pain been in the **last 2 weeks**?

Not at all	Slightly	Moderately	Very much	Extremely
☐	☐	☐	☐	☐
