# Supplementary figures and images for: Societal costs of older adults with low back pain seeking chiropractic care: findings from the BACE-C cohort study
Source: Chiropr Man Therap. 2024 Nov 6;32:31. doi: 10.1186/s12998-024-00553-0 (PMC11539272; doi:10.1186/s12998-024-00553-0)

**APPENDIX 2**

Figure 1. Societal costs throughout one year of all participants.


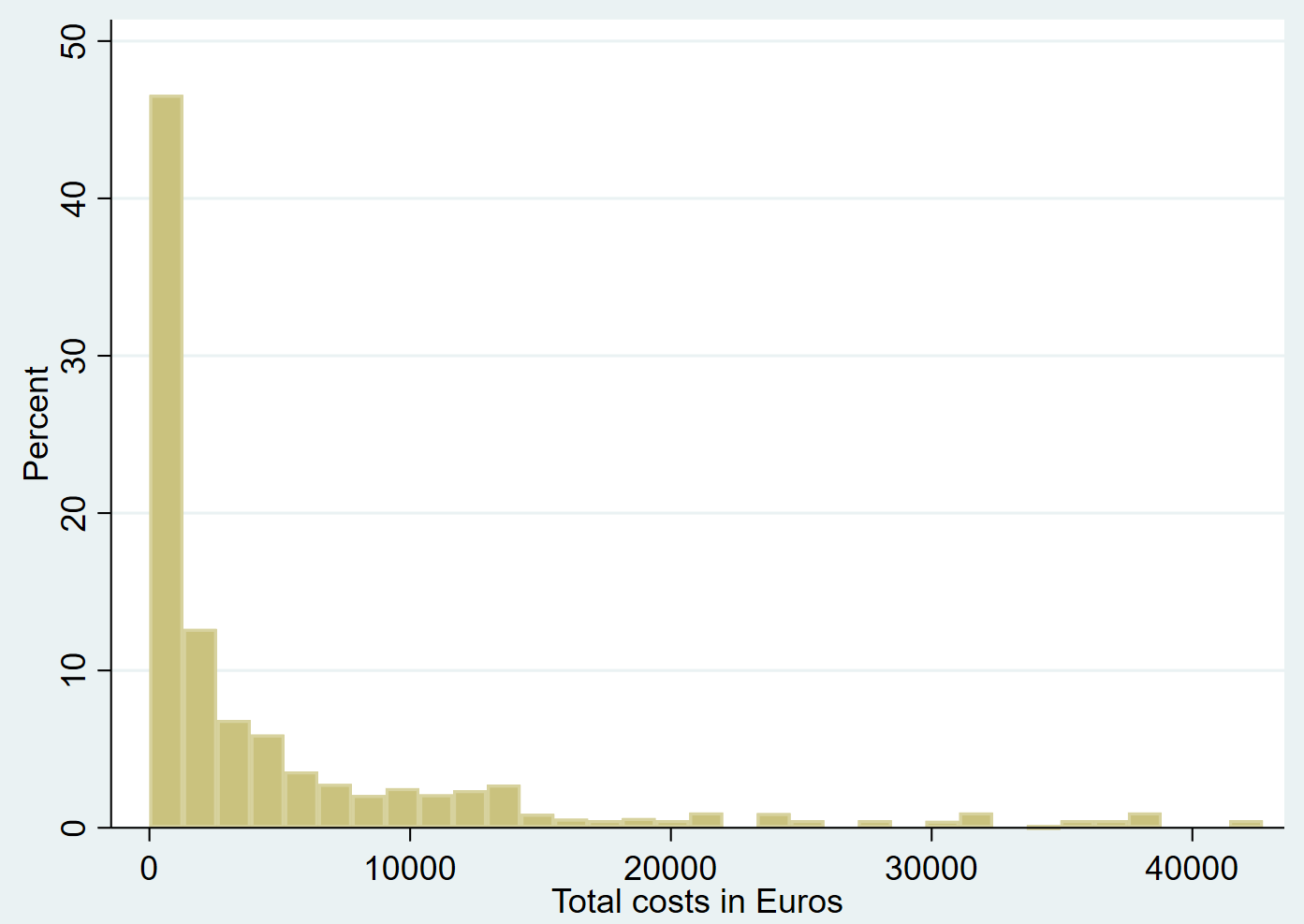

Supplement: Supplementary file 3 — Additional file 3. [file 12998_2024_553_MOESM3_ESM.docx]
