# Supplementary material for: Societal costs of older adults with low back pain seeking chiropractic care: findings from the BACE-C cohort study
Source: Chiropr Man Therap. 2024 Nov 6;32:31. doi: 10.1186/s12998-024-00553-0 (PMC11539272; doi:10.1186/s12998-024-00553-0)
Supplement: Supplementary file 4 — Additional file 4. [file 12998_2024_553_MOESM4_ESM.docx]

# **Appendix 4**

Figure 1. Societal costs divided by 3-month time frames

Figure 2. Numeric Rating Scale Pain scores over one year – All participants
