# Supplementary material for: Societal costs of older adults with low back pain seeking chiropractic care: findings from the BACE-C cohort study
Source: Chiropr Man Therap. 2024 Nov 6;32:31. doi: 10.1186/s12998-024-00553-0 (PMC11539272; doi:10.1186/s12998-024-00553-0)
Supplement: Supplementary file 5 — Additional file 5. [file 12998_2024_553_MOESM5_ESM.docx]

Appendix 5.

**Univariate analyses for the pensioners**

|  | **Odds ratio** | **95% CI** | **p-value** |
| --- | --- | --- | --- |
| **BMI** | 0.97 | 0.86-1.10 | 0.67 |
| **LBP intensity (0-10)** | 1.04 | 0.79-1.37 | 0.75 |
| **Age** | 0.95 | 0.84-1.06 | 0.34 |
| **Back-related functioning (0-24)** | 1.06 | 0.96-1.18 | 0.21 |
| **QALY** | 2.29 | 0.36-14.42 | 0.38 |
| **Symptom duration** | 0.97 | 0.89-1.06 | 0.52 |
| **Gender** | 1.53 | 0.48-4.93 | 0.47 |
| **Education** | 1.04 | 0.55-1.98 | 0.89 |
| **Civil status** | 1.81 | 0.45-7.19 | 0.40 |
| **Previous episode of LBP** | 1.01 | 0.39-2.66 | 0.97 |
| **Radiating pain** | 0.96 | 0.47-1.97 | 0.91 |
| **Alcohol use** | 0.87 | 0.57-1.32 | 0.53 |
| **Global Perceived effect** | 0.52 | 0.43-6.15 | 0.60 |
| **Overall Physical activity** | 0.99 | 0.99-1.00 | 0.68 |
| **Comorbidity (Y/N)** | 1.06 | 0.20-5.67 | 0.94 |
| **StarT Back grouping** | 0.97 | 0.18-5.38 | 0.97 |
